# Influence on interradicular bone volume of Invisalign treatment for adult crowding with interproximal enamel reduction: a retrospective three-dimensional cone-beam computed tomography study

**DOI:** 10.1186/s12903-018-0569-4

**Published:** 2018-06-08

**Authors:** Andreas Hellak, Nicola Schmidt, Michael Schauseil, Steffen Stein, Thomas Drechsler, Heike Maria Korbmacher-Steiner

**Affiliations:** 1 Department of Orthodontics, University Hospital Giessen and Marburg, Campus Marburg, Georg-Voigt-Strasse 3, 35039 Marburg, Germany; 2 Private practice, Wiesbaden, Germany; 3 Abt. für Kieferorthopädie, UKGM Standort Marburg, Georg-Voigt-Strasse 3, 35039 Marburg, Germany

**Keywords:** Aligner, Adult crowding, Interradicular bone volume, IER, Bone quantity, CBCT

## Abstract

**Background:**

The aim of this study was to use three-dimensional datasets to identify associations between treatment for adult crowding, using Invisalign aligner and interproximal enamel reduction (IER), and changes in the volume of interradicular bone.

**Methods:**

A total of 60 cone-beam computed tomography (CBCT) scans from 30 adult patients (28 women, two men; 30 CBCTs pre-treatment, 30 post-treatment) were examined retrospectively in order to measure bone volume three-dimensionally. The patients’ average age was 36.03 ± 9.7 years. The interradicular bone volume was measured with OsiriX at four levels in the anterior tooth areas of the maxilla and mandible. Differences in bone between T0 and T1 were analyzed with IBM SPSS 21.0 using the Wilcoxon test for paired samples.

**Results:**

Overall, a slight increase in the quantity of bone was found (0.12 ± 0.73 mm). There was a highly significant increase in bone in the mandible (0.40 ± 0.62 mm; *P* <  0.001), while in the maxilla there was a slight loss of bone, which was highly significant in the apical third (− 0.16 ± 0.77 mm; *P* = 0.001).

**Conclusions:**

Overall, treatment for adult crowding using an aligner and IER appears to have a positive effect on interradicular bone volume, particularly in patients with severe grades of the condition (periodontally high-risk dentition). This effect is apparently independent of IER. This is extremely important with regard to the treatment outcome, since IER and root proximity have been matters of debate in the literature and teeth should remain firmly embedded in their alveolar sockets.

## Background

Among adult patients there is growing interest in having a functionally healthy and aesthetically attractive dentition [1]. Patients often have adult crowding and wish to have malpositioning corrected as invisibly as possible [2, 3]. There are many treatment options e.g. using aligners and interproximal enamel reduction (IER).

One possible treatment for relieving crowding consists of expanding the dental arch in the labial direction in order to provide space for normal positioning of the affected teeth. Another method of creating space is IER. Potential periodontal changes in the anterior tooth area during orthodontic treatment with IER for adult crowding have been a topic of discussion in the literature [4–6]. In addition to the treatment of patients with periodontally healthy dentition, the question arises for the orthodontist of the way in which periodontally high-risk dentition is likely to behave during treatment. Vermylen et al. [7] defined an interradicular distance of 0.8 mm or less as root proximity and a risk marker for periodontal disease.

There have to date been no three-dimensional investigations of changes in interradicular bone volume in relation to treatment for adult crowding. The present study was carried out in order to investigate whether orthodontic treatment and resolution of crowding may even lead to an improvement in the bone situation. As a result of the use of conventional two-dimensional imaging to date, only limited quantification of the pre-therapeutic and post-therapeutic interradicular bone situation has been possible [8]. It is only modern three-dimensional cone-beam computed tomography (CBCT) scanning that has made it possible to carry out 3D analysis of the bone structures and the way in which they respond to tooth movements [9, 10, 11]. The aim of the present study was to investigate whether and to what extent orthodontic treatment with Invisalign aligners and IER leads to a change in the interradicular bone volume. Specifically, the following questions were addressed:

- How is the interradicular bone volume altered by aligner therapy?
- What effects on interradicular root distances are associated with interproximal enamel reduction (IER)?
- In what ways does the interradicular bone volume change after initial findings corresponding to a root proximity (=interradicular distance of ≤0.8 mm, a so called risk marker for periodontal disease [4])?

## Methods

Changes in the interradicular distance were measured at a total of 720 measurement points in the present study. Pre-therapeutic and post-therapeutic cone-beam computed tomography (CBCT) scans from a total of 30 patients (28 women, two men) were examined retrospectively. In accordance with the SEDENTEXCT guidelines, the CBCT scans were taken for two reasons:

1. For periodontal assessment (a total of 26 cases). These patients had a fragile gingival type, with less bone in the anterior tooth area. Pre-treatment CBCT was therefore intended to visualize root proximity and resorption and help with therapeutic decision-making on whether to carry out IER or extraction of one anterior tooth. The following CBCT was intended to visualize root proximity and resorption after the completion of possible treatment, to check whether IER was still appropriate for creating space, since the teeth need to be covered by bone in order to avoid recession.
2. For temporomandibular joint assessment (a total of four cases). Some patients had craniomandibular disorders (CMD) with rheumatoid arthritis. In this small number of selected cases, pre-treatment CBCT was used to identify bone degenerative deformity, condylar positions, and bony structures in the temporomandibular joint. After successful CMD therapy and orthodontic treatment, CBCT scans were taken again to view the condylar positions and bony structures in the temporomandibular joint due to recurrent CMD problems and in order to adjust anti-inflammatory therapy.

The patients’ average age was 36.03 ± 9.7 years. The use of the data was approved by the ethics committee of Marburg University Hospital (ref. no. 34/15).

The following inclusion and exclusion criteria were applied. Inclusion criteria:

- Presence of adult crowding capable of being adjusted using conservative orthodontic space-gaining measures such as protrusion, proclination, expansion and IER
- Permanent dentition
- Successfully completed treatment with Invisalign aligners
- Availability of one CBCT each from before and after treatment

The following parameters represented exclusion criteria:

- Extraction of anterior teeth during the course of treatment
- macrodontia/ hypoplasia
- abnormal change in tooth morphology
- Prosthetic treatment
- Skeletal anomalies
- General medical findings relevant to bone metabolism (e.g., osteoporosis, dysostosis, etc.)
- Periodontal disease and previous periodontal surgery procedures

All of the images were taken with a KaVo 3D eXam DVT system (KaVo Dental Ltd., Biberach an der Riss, Germany) using a scan with 360° revolution, a duration of 26.9 s (X-ray source voltage: 120 kVp; X-ray source current: 5 mA) and a voxel size of 0.25 mm. The datasets were collected and evaluated using OsiriX (Pixmeo, Bernex, Switzerland) with an Apple OS X operating system.

All of the patients had provided written consent to the use of their data in the study (in accordance with the Helsinki Declaration). The data were all analyzed on a semi-blinded basis.

### Measurement of interradicular bone volume

**Fig. 1:**
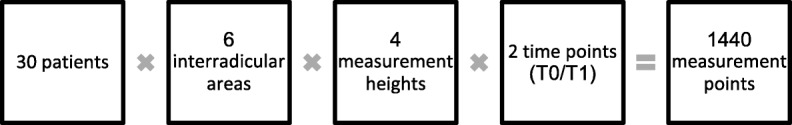
Total measurement data

**Fig. 2:**
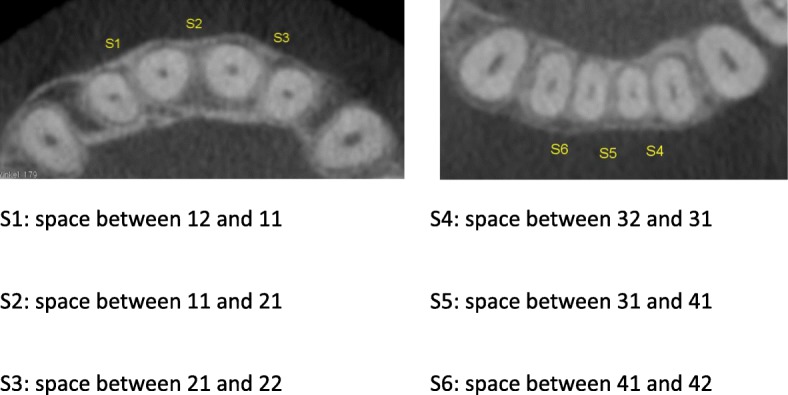
Measurement points for interradicular distances

**Fig. 3:**
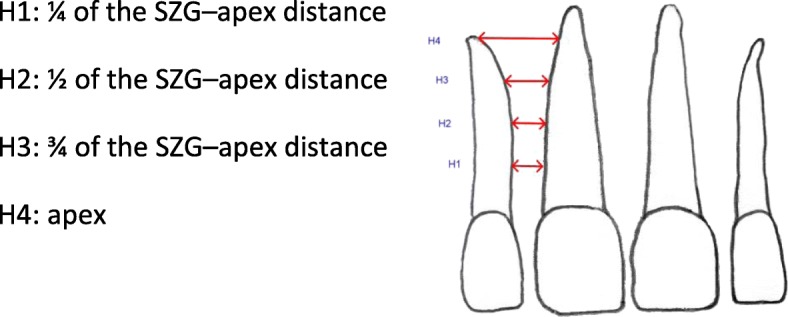
Two-dimensional diagram showing the measurement distances used to determine interradicular distances

Measurements of the mesiodistal interradicular distance (Fig. 1) were modified in the OsiriX DICOM viewer using the method described by Sawada et al. [7]. The six interradicular areas between the lateral incisors in the maxilla and mandible were measured (Figs. 2 and 3).

**Fig. 4:**
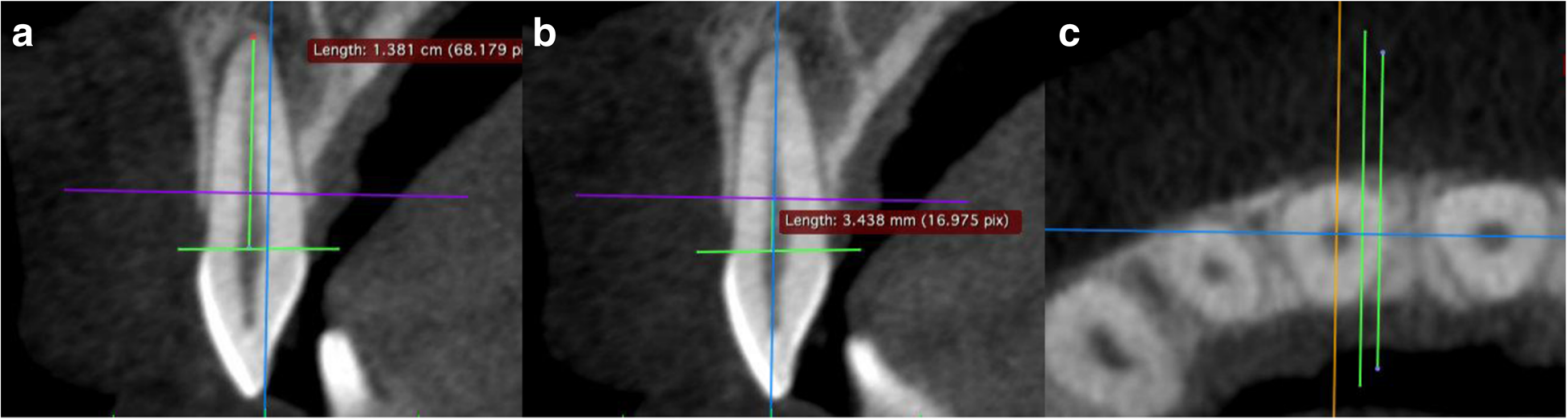
Sagittal plane. **a** Measurement of the length of the apex to enamel–cement boundary (ECB) distance. **b** Setting the first measurement level, H1, at one-quarter of the ECB–apex distance. **c** Creating the auxiliary lines parallel to the sagittal axis in the axial view

The shorter of the roots in the two teeth adjoining the interradicular space was set as the reference tooth. A connecting line was drawn in the sagittal view from the buccal enamel–cement boundary (ECB) to the palatal or lingual enamel–cement boundary (Fig. 4). From the intersection of that line with the center of the root canal, the length to the apex was measured parallel to the dental axis/coronal plane (the ECB–apex distance), and the tooth was divided into four equal-sized sections. This resulted in the four measurement levels (Fig. 3). The sagittal view was used to adjust the axial plane to the desired height (Fig. 4).

**Fig. 5:**
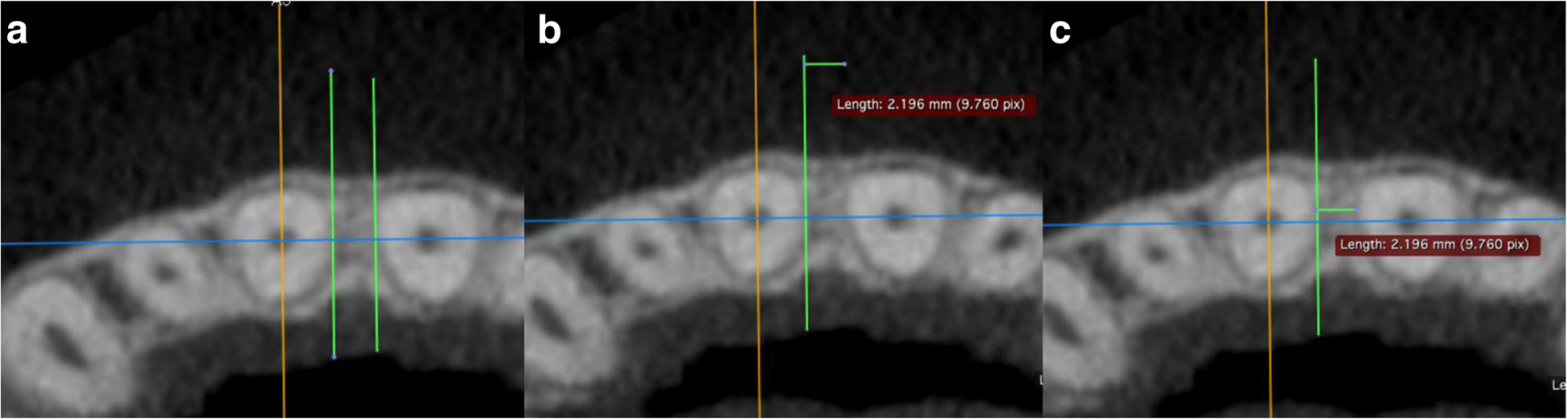
Measurement of mesiodistal interradicular distance S1 at the H1 level on the axial plane. **a** Shifting of the auxiliary lines to the root surfaces. **b** Measuring the shortest interradicular distance by shifting an auxiliary line. **c** Display of the measurement distance

The interradicular distance was measured in the axial view. For this purpose, two auxiliary lines were drawn parallel to the sagittal plane and were shifted in parallel as far as the root surfaces of the neighboring teeth (Fig. 5). In the measurement levels set, the interradicular distance was thus measured as the shortest distance between the root surfaces.

Whether and to what extent IER or expanding the dental arch in the labial direction influenced the interradicular space was investigated using ClinCheck software with the exact IER protocol. For each interradicular space we analyzed the change of the distance between the roots in comparison to the amount of IER. The total amount of IER was between 0.0 mm and 0.5 mm. In our study crowding was defined as the difference in millimeter between the arch perimeter and the mesial to distal tooth size total form S1-S3 (upper anterior jaw) and S4-S6 (lower anterior jaw) (Fig. 2). Maxillary and mandibular arches were classified separately. Each case was classified as presenting mild discrepancy crowding between − 0.1 mm to − 5 mm according to Proffit. W. R. and H. W. Field. *Contemporary Orthodontics*. St Louis, Mo: Mosby; 2000:224.

All of the digital volume tomograms were analyzed a second time by the same investigator (N.S.) one month later, to allow assessment of the reproducibility of the measurements. The means of the two evaluations were used for statistical analysis. All patients were treated by one operator (T.D.) in the same office.

Statistical analyses were carried out using IBM SPSS for Mac, version 21.0 (IBM Corporation, Armonk, New York, USA). The intraoperator correlation for each examination was initially calculated. For further analysis, the normal distribution of the values was checked. The values were tested for significant differences using the Wilcoxon test. The significance level was set at *P* = 0.05.

## Results

Kendall’s tau-b test showed a highly significant (*P* <  0.001, two-sided) intraoperator correlation (*r* = 0.837) for the interradicular distance measurements.

Increases in the interradicular distance were observed in the mandible, and decreases were observed in the maxilla (Table 1). The increase in interradicular space in the mandible was greater than the loss of space in the maxilla. The Wilcoxon test showed highly significant (*P* ≤ 0.001) changes between T0 and T1 at all levels in the mandible, highly significant (*P* ≤ 0.01) changes at the apex measurement level, and significant (*P* ≤ 0.05) changes at the three-quarter level in the maxilla (Table 1).

**Table 1:** Descriptive comparison of differences in the interradicular distance measurements between T1 and T0, using the Wilcoxon test^a^ for statistical analysis

Measurement level		n	Minimum	Maximum	Mean	SD	Wilcoxon test^a^	T1–T0
Maxilla								
¼	T1–T0	90	−1.75	1.27	−0.07	0.52	Z	−1.189 ^b^
							A. significance (two-sided)	0.234
½	T1–T0	90	−2.40	1.33	−0.03	0.66	Z	−0.127 ^b^
							A. significance (two-sided)	0.899
¾	T1–T0	90	−2.45	1.67	−0.22	0.75	Z	−2.505 ^b^
							A. significance (two-sided)	0.012
Apex	T1–T0	90	−3.02	1.94	−0.32	1.03	Z	−2.565 ^b^
							A. significance (two-sided)	0.01
Mandible								
¼	T1–T0	90	−0.68	1.35	0.30	0.46	Z	−5.237 ^c^
							A. significance (two-sided)	< 0.001
½	T1–T0	90	−0.87	1.71	0.42	0.52	Z	−6.113 ^c^
							A. significance (two-sided)	< 0.001
¾	T1–T0	90	−1.33	2.49	0.45	0.62	Z	−6.051 ^c^
							A. significance (two-sided)	< 0.001
Apex	T1–T0	90	−1.86	2.60	0.40	0.84	Z	−4.048 ^c^
							A. significance (two-sided)	< 0.001

*SD* standard deviation

^a^Wilcoxon signed rank test

^b^Based on positive ranks

^c^Based on negative ranks

### Effects of IER

A positive effect was noted after treatment in 62.5% of all interdental spaces in which IER was carried out; however, the distance decreased in 37.5%. The effect was almost identical without IER (Table 2). Overall, it was found that IER did not have any statistically significant effects on the changing interradicular space conditions.

**Table 2:** Effects of interproximal enamel reduction on the interradicular distance in 180 interproximal spaces

	With IER (*n* = 104)	Without IER (*n* = 76)
Interradicular distance increased	62.50%	63.16%
Interradicular distance decreased	37.50%	36.84%

*IER* interproximal enamel reduction

### Periodontally critical situation (interradicular distance ≤0.8 mm)

In all, 17.2% of the pre-therapeutic interradicular measurement points had an interradicular distance ≤0.8 mm (Table 3), and the majority of these were in the mandible. As Table 3 shows, the treatment had a positive effect, since afterwards only 7.9% of the measurement points still had an interradicular distance ≤0.8 mm.

**Table 3:** Interradicular distance ≤0.8 mm at time points T0 and T1 (*n* = 720)

	T0	T1
Maxilla	1.53%	1.11%
Mandible	15.69%	6.81%
Total	17.22%	7.92%

It was then investigated whether a periodontally high-risk dentition (≤ 0.8 mm) benefited more from aligner treatment than a periodontally healthy dentition (> 0.8 mm). Of the 124 measurement points that had a root distance ≤0.8 mm in the initial findings, 88.71% had increased space after treatment. An interradicular space increase of more than 0.8 mm was even observed in 71.77% (Table 4). An increase in space of more than 0.8 mm was observed in nine of 11 measurement points in the maxilla (81.82%), and in 80 of 113 measurement points in the mandible (70.8%).

**Table 4:** Increase in the interradicular distance between T0 and T1 of periodontally high-risk dentition, including interradicular space increases to > 0.8 mm

*n* = 124 (≤ 0.8 mm)	Increases in space	Increases in space > 0.8 mm
Maxilla	90.91%	81.82%
Mandible	88.5%	70.80%
Total	88.71%	71.77%

By comparison, periodontally high-risk dentitions showed much larger increases in the interradicular bone volume (Table 5). As Table 5 shows, the result was highly significant statistically (*P* ≤ 0.001).

**Table 5:** Descriptive statistics for interradicular changes, classified into groups with a root proximity (periodontally high-risk dentition - interradicular bone quantity at T0 ≤ 0.8 mm) or with a periodontally normal dentition (interradicular bone volume at T0 >  0.8 mm) with Wilcoxon signed rank test for statistical analysis

Distance		n	Min.	Max.	Mean	SD	Wilcoxon test (≤ 0.8 mm vs. > 0.8 mm)	
≤ 0.8 mm	T1–T0	124	−0.45	2.49	0.60	0.54	Z	−8.071
> 0.8 mm	T1–T0	596	−3.02	2.60	0.02	0.75	A. significance (two-sided)	< 0.001

*SD* standard deviation

## Discussion

In the group of patients investigated in the present study, treatment for adult crowding was associated with an overall increase in interradicular space. The increased space was gained particularly in the mandible, as the increase was larger than the slight loss of space in the maxilla. One possible explanation for this might be the varying severity of the crowding. In this group of patients, adult crowding usually appeared earlier and with greater severity in the mandible than in the maxilla. More extensive measures to create space were therefore needed with a smaller bone volume. Another explanation might be the different methods used to create space.

One possible treatment for relieving crowding consists of expanding the dental arch in the labial direction in order to allow space for normal positioning of these teeth [12]. This method of space creation was used much more often in the mandible. The positive effect of reshaping the dental arch thus appears to have a strong influence on increases in interradicular space.

Another method of creating space is IER [13]. Although the roots ought to move closer to each other after the removal of enamel during IER, the positive effect of reshaping the dental arch appears to outweigh this, at least in the mandible. This increase in space despite IER has also been confirmed in other studies. In two-dimensional studies, Zachrisson et al. [4] found that due to crowding, the roots have to be closer together than in correctly aligned teeth.

During treatment for adult crowding, interproximal enamel reduction (IER) was only carried out supportively in a few interdental spaces. When only the subtopic of IER is considered, it is notable that IER did not have any significant effect on the bone volume between the anterior dental roots. The distribution pattern of changes in the interradicular distance was almost identical with and without IER.

In general, the advantage of interproximal enamel reduction is that the extent of the expansion in the labial direction can be reduced, thereby reducing the risk of bone dehiscence occurring. In addition, the widened approximal contacts stabilize the treatment result [14]. These findings are also consistent with those reported in the clinical studies by Zachrisson et al. [4], in which no deterioration was observed on dental film more than 10 years later after approximal enamel reduction. However, precise three-dimensional measurement of interradicular spatial conditions was not possible due to the use of two-dimensional radiographic diagnosis in the study. An improvement in the aesthetic appearance can also be expected as a result of relieving anterior crowding, due to the avoidance of what are known as “black triangles.” The creation of optimal apposition areas for the gingiva also reduces or prevents retrusion of the interdental papillae [15].

The most noticeable positive effect was seen when teeth with root proximity were treated. Vermylen et al. [7] defined 0.8 mm or less bone or interdental tissue as representing root proximity. Interdental spaces of this size are poorly accessible for periodontal treatment and are less able to resist periodontal disease [16]. Radicular distances with this potentially poor initial condition showed improvement in the spatial situation in approximately 89% of cases, and improvement beyond the critical range (> 0.8 mm after treatment) in approximately 72%. This means that a periodontally high-risk dentition benefited more from the aligner treatment than a periodontally healthy dentition. However, it must be mentioned here that root proximity is not the cause of periodontal disease, but only represents a risk factor [17–20]. Bacterial plaque is one of the main causes of periodontal inflammation [21].

Another advantage of the present study is the three-dimensional imaging of the interradicular spaces. Other studies on measurement of bone volume have only been carried out using two-dimensional images, and have always noted the difficulty of depicting the interradicular distance precisely [8]. Two-dimensional images are also known as cumulative images. As a result of the cumulation, superimposed roots in crowded conditions are difficult to distinguish and the spaces are difficult to measure, due to differing enlargement factors. This lost information can be displayed in three-dimensional images [22]. For CBCT analysis, the question arises of whether the image resolution is sufficient to allow precise analysis. Gribel et al. [23] compared CBCT measurements with direct measurements of dry skulls. They found that CBCT scanning with a slice thickness of 0.3 mm was extremely precise, with a mean deviation of the measurements from the direct measurements of 0.1 mm.

Unfortunaly this study has a retrospective design with a risk of bias. A prospective randomized controlled trial would be interesting, but could currently not be carried out because of the ALARA principle. In view of the principle that radiation exposure should be “as low as reasonably achievable” (ALARA), CBCT is not indicated as a routine method for the imaging of bone support [24, 25]. A CBCT may only be indicated in selected cases in which clinical and conventional examinations do not provide the information needed for treatment. The operator always needs to consider its use carefully. If a CBCT is needed the use of shorter scans and a reduced effective radiation dose is recommended [26, 27]. A study group with a more balanced sex ratio would be desirable, because most of the patients included in this study were women. This is due to the retrospective character of our study. However, presenting these data from the context of aligner treatment for adult crowding and possible interradicular bone changes may be helpful. Further research and additional information on the topic would be desirable.

## Conclusions

Overall, treatment of adult crowding using Invisalign and IER, particularly in patients with severe conditions (with periodontally high-risk dentition), appears to have a positive effect on the interradicular bone volume, at least in adult female patients. The effect is also apparently independent of IER.

## Abbreviations

ALARA: As low as reasonably achievable
CBCT: Cone beam computed tomography scans
IER: Interproximal enamel reduction
